# Strategic wound and nutritional management for short bowel syndrome and wound dehiscence following non-occlusive mesenteric ischemia: a case report

**DOI:** 10.1097/RC9.0000000000000913

**Published:** 2026-09-02

**Authors:** Kenichiro Yambe, Shingo Tsujinaka, Yoh Kitamura, Tomoya Miura, Kei Nakagawa, Toru Nakano

**Affiliations:** a Division of Hepato-Biliary and Pancreatic Surgery, Tohoku Medical and Pharmaceutical University, Sendai, Japan; b Division of Gastroenterologic Surgery, Tohoku Medical and Pharmaceutical University, Sendai, Japan

**Keywords:** case report, negative-pressure wound therapy, non-occlusive mesenteric ischemia, nutritional support, short bowel syndrome, wound dehiscence

## Abstract

**Introduction and importance:**

Non-occlusive mesenteric ischemia (NOMI) is life-threatening and may require extensive bowel resection, resulting in short bowel syndrome (SBS), nutritional deficiencies, and impaired wound healing. Severe wound dehiscence further complicates postoperative management, and reports describing this sequence are limited. This report highlights coordinated, phase-oriented management in an immunocompromised patient.

**Presentation of case:**

An 81-year-old female receiving immunosuppressive therapy underwent emergency Hartmann’s procedure for perforated sigmoid diverticulitis. Postoperative septic shock required vasopressor support. On postoperative day (POD) 9, NOMI was diagnosed, necessitating subtotal small bowel resection and total colectomy, leaving approximately 70 cm of jejunum with an end jejunostomy. Extensive wound dehiscence developed by POD 21. Management included debridement, povidone-iodine sugar paste, 4 weeks of negative-pressure wound therapy with instillation, and trafermin. Complete secondary-intention closure without skin grafting was achieved by POD 90. Enteral and oral nutrition were gradually introduced while parenteral support was continued, and the high-output stoma was controlled with antidiarrheal agents and proton-pump inhibitors. The patient was transferred for rehabilitation on POD 120. At 15 months, she remained well, tolerated a regular diet, and required parenteral nutrition only on weekends.

**Clinical discussion:**

Delayed NOMI should be considered after septic shock despite transient hemodynamic stabilization. Phase-oriented wound care, staged nutritional escalation, and stoma output control supported secondary-intention healing without reconstructive surgery in a patient with SBS-related malnutrition.

**Conclusion:**

Coordinated wound and nutritional management may enable sustained recovery after NOMI complicated by SBS, high-output stoma, and extensive wound dehiscence.

## Introduction

Non-occlusive mesenteric ischemia (NOMI) causes intestinal ischemic necrosis without organic occlusion of the main trunk vessels. It typically occurs in the context of heart failure, septic shock, and vasopressor use, with a mortality rate exceeding 50%^[^1^]^. In NOMI survivors, short bowel syndrome (SBS) may develop following extensive resection; approximately 49% of adult patients with SBS and residual small bowel length <100 cm remain dependent on parenteral nutrition (PN)^[^2^]^ at 2 years postoperatively. Moreover, nutritional and trace element deficiencies further complicate wound healing^[^3^]^.

HIGHLIGHTS

- Non-occlusive mesenteric ischemia followed surgery for perforated diverticulitis.
- Extensive bowel resection caused short bowel syndrome and a high-output stoma.
- Staged wound care included debridement, negative-pressure therapy, and trafermin.
- Staged nutrition promoted healing despite short bowel syndrome-related malnutrition.
- Coordinated wound and nutritional care enabled healing without reconstructive surgery.

The incisional surgical site infection (SSI) rate is as high as 82.4% in cases where primary wound closure is performed for colonic perforation with generalized peritonitis^[^4^]^, and severe SSI can lead to wound dehiscence. Additionally, 13.5% of surgically treated patients with NOMI develop SSI^[^5^]^. In such clinical settings, NOMI, SBS, and severe SSI may occur sequentially; however, to our knowledge, reports of cases complicated by all three are scarce. We report a case of severe SSI complicated by SBS following NOMI that was successfully managed with intensive wound care, including negative-pressure wound therapy (NPWT) and staged nutritional strategies. To our knowledge, the sequential occurrence of perforated diverticulitis, septic shock, delayed intestinal ischemia, massive bowel resection, multidrug-resistant wound contamination, and severe wound dehiscence in a single patient has rarely been reported. The coordinated, phase-oriented management required in this case is instructive for surgeons and intensivists. This case is reported in accordance with the Surgical Case Report (SCARE) guidelines^[^6^]^.

## Presentation of case

### Initial presentation and Hartmann’s procedure

An 81-year-old woman presented with worsening abdominal pain and vomiting. Five days before admission, she developed abdominal pain and diarrhea and was diagnosed with gastroenteritis at a local clinic. On the morning of admission, after oral intake she again developed vomiting and abdominal pain, which persisted and prompted a nighttime visit to our emergency department.

Her medical history included hypertension, dyslipidemia, lumbar disc herniation, and polymyalgia rheumatica. Her medications included prednisolone (3 mg/day), iguratimod (50 mg/day), and methotrexate (4 mg/day). On arrival, despite stable vital signs, the physical examination revealed diffuse abdominal tenderness, with percussion tenderness, guarding, and rebound tenderness.

Laboratory tests showed no significant abnormalities except for the arterial blood gas analysis, which revealed mild metabolic acidosis with elevated lactate (3.3 mmol/L). Contrast-enhanced computed tomography (CT) revealed free intra-abdominal air, a large amount of ascites, and suspected fecal leakage around the sigmoid colon (Fig. 1A). The patient was diagnosed with generalized peritonitis due to sigmoid colon perforation and underwent emergency Hartmann’s procedure. Intraoperatively, full-thickness necrosis of the sigmoid colon and fecal leakage were observed (Fig. 1B). Given her advanced age, chronic immunosuppression, generalized fecal peritonitis, full-thickness sigmoid colon necrosis, and intraoperative vasopressor requirement, Hartmann’s procedure was selected to achieve definitive source control while avoiding a high-risk primary anastomosis. The pathological findings were consistent with perforation of a sigmoid colon diverticulum (Fig. 1C). Postoperatively, the patient developed septic shock requiring vasopressor support and was admitted to the intensive care unit (ICU). A mean arterial pressure of approximately 65 mmHg was maintained with noradrenaline up to 0.3 µg/kg/min and vasopressin at 2 U/h; vasopressor requirements peaked on postoperative days (PODs) 1–2, while lactate levels remained 2–3 mmol/L. Broad-spectrum antibiotics (meropenem and vancomycin) and a stress dose of prednisolone (10 mg/day) were also administered. The patient became vasopressor-free by POD 6 and was discharged from the ICU.

**Figure 1.**
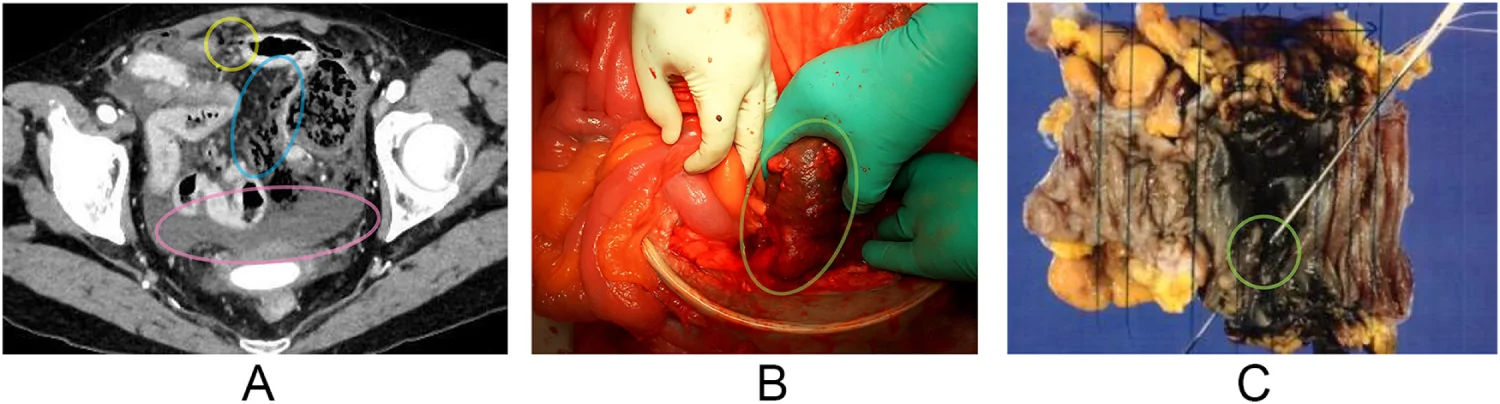
Imaging, intraoperative, and specimen findings at the onset of a sigmoid colon perforation. (A) Contrast-enhanced computed tomography shows free intraperitoneal air (yellow circle), extraluminal fecal material (blue circle), and a large volume of ascites (pink circle), consistent with a perforated sigmoid diverticulum. (B) Intraoperative view shows full-thickness necrosis of the sigmoid colon with gross fecal contamination (green circle). (C) Resected specimen confirming a perforated sigmoid diverticulum (green circle).

### Re-laparotomy due to NOMI

However, on POD 8, the patient developed vomiting and abdominal distension. Repeat contrast-enhanced CT on POD 9 showed segmental areas of poor enhancement (Fig. 2A), although the abdomen remained soft with minimal tenderness, and laboratory data were stable. However, necrosis was observed at the midline and of the peristomal skin (Fig. 2B). Several hours later, massive amounts of intestinal fluid were drained from the midline wound, and an emergency re-laparotomy was performed.

**Figure 2.**
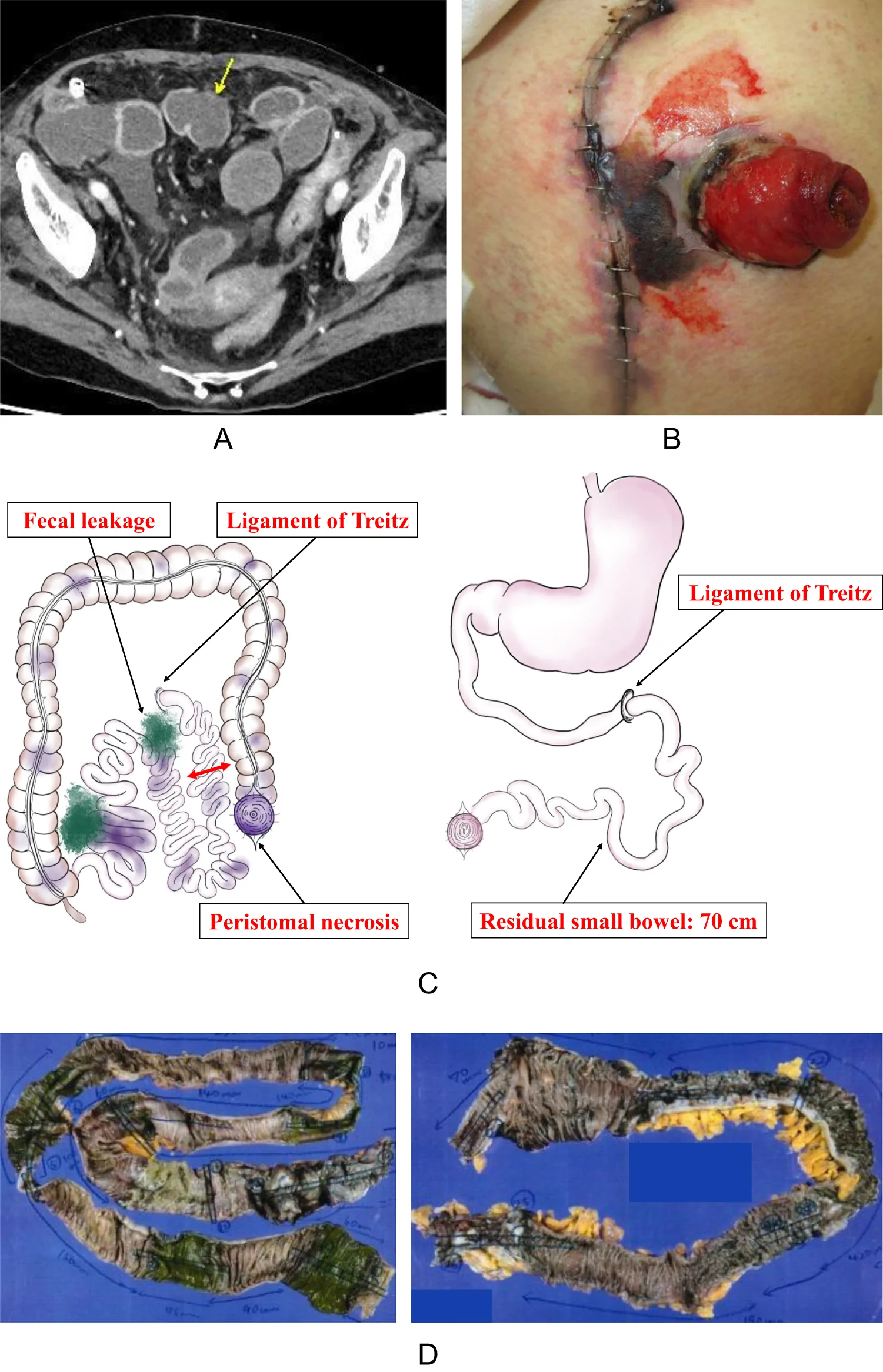
Imaging, intraoperative, and specimen findings at the onset of non-occlusive mesenteric ischemia. (A) Contrast-enhanced computed tomography showing segmental areas of poor enhancement in the small intestine (yellow arrow), suggestive of intestinal ischemia. (B) Skin necrosis at the midline wound and peristomal area. (C) Left: Schematic illustration of segmental and discontinuous intestinal ischemia or necrosis with fecal contamination and their anatomical relationship to the ligament of Treitz. Right: Schematic illustration of the anatomy after intestinal resection, leaving approximately 70 cm of the jejunum with an end jejunostomy. (D) Gross specimens showing extensive segmental ischemic changes in the resected bowel. Left: small intestine. Right: terminal ileum and entire colon.

During re-laparotomy, extensive segmental and discontinuous ischemia with necrosis was observed in the small intestine and the remaining colon. Pulsation of the superior mesenteric artery was preserved, with no main trunk occlusion, consistent with NOMI. Subtotal small bowel resection and total colectomy were performed, leaving approximately 70 cm of jejunum from the ligament of Treitz, and an end jejunostomy was created (Fig. 2C). Necrotic abdominal wall, skin, and subcutaneous tissue were debrided. The macroscopic appearance (Fig. 2D) demonstrated extensive ischemic changes, and pathological findings predominantly involving peripheral vessels were consistent with NOMI.

### Wound management

The postoperative wound course, timeline, and nutritional management are summarized in Fig. 3 and Supplemental Digital Content Table 1, available at: https://links.lww.com/IJSCR/A139. Beginning around POD 12, wound exudate increased. Wound dehiscence began around POD 15 and progressed to extensive dehiscence by POD 21, involving the skin and subcutaneous tissue while preserving the fascial layer. The plastic surgery service and the in-hospital wound care team were consulted. Necrotic tissue was repeatedly debrided, and povidone-iodine sugar paste was applied for wound bed cleansing. On POD 29, NPWT with instillation (V.A.C. ULTA Therapy System; KCI, an Acelity company, San Antonio, TX, USA) was initiated and continued for 4 weeks. After NPWT discontinuation, exudate decreased remarkably and epithelialization progressed, although biofilm removal with povidone–iodine sugar paste was required several times. By POD 69, adequate granulation and wound contraction were achieved, and trafermin (basic fibroblast growth factor) spray was applied to further promote healing. Wound cultures yielded multiple multidrug-resistant organisms, including methicillin-resistant *Staphylococcus aureus* and extended-spectrum *β*-lactamase-producing *Escherichia coli*, for which targeted antimicrobial therapy was administered as necessary. Complete wound closure was achieved by POD 90.

**Figure 3.**
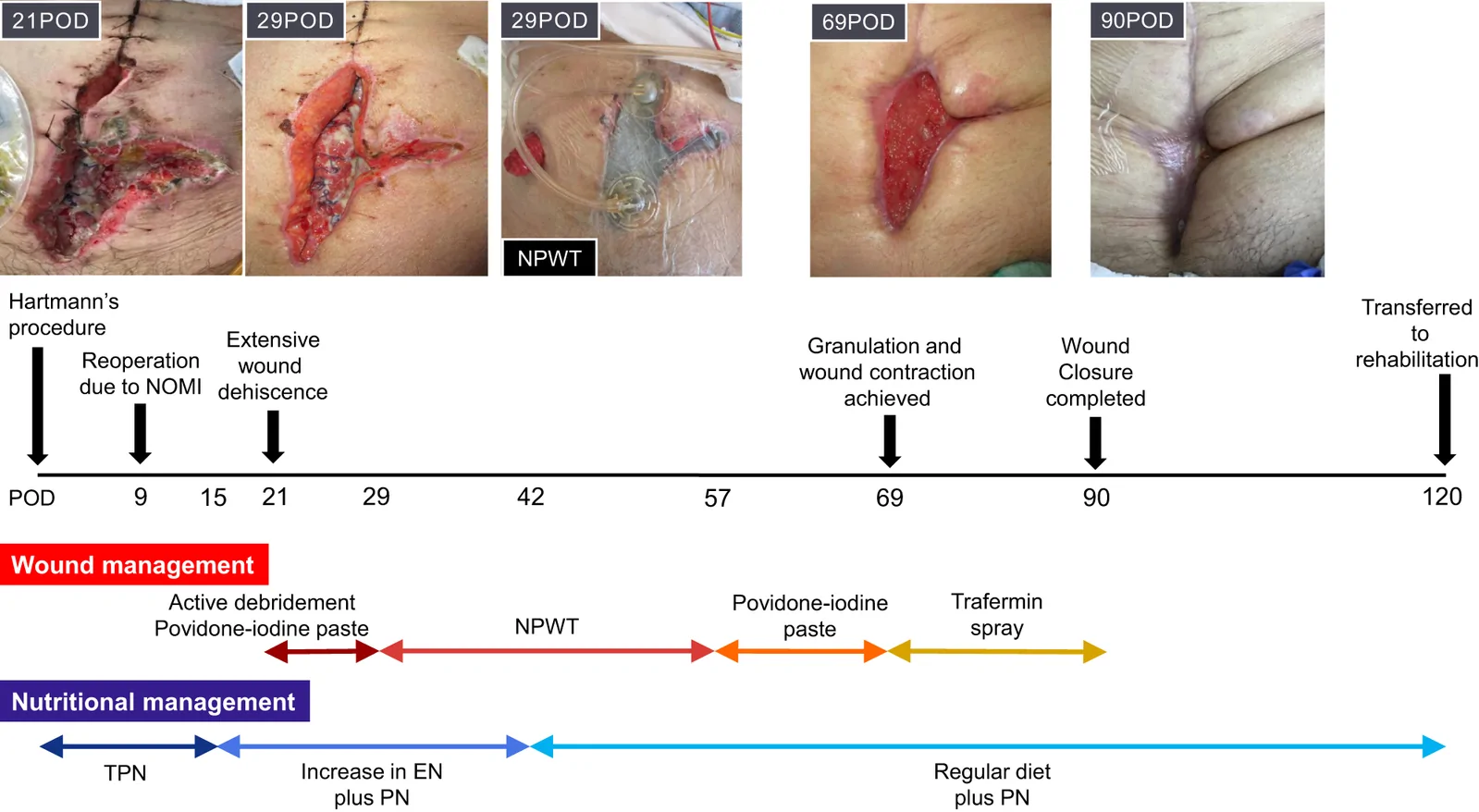
Postoperative wound course and timeline of wound and nutritional management. Representative wound images demonstrate extensive wound dehiscence (postoperative day 21), persistent wound dehiscence (postoperative day 29), negative-pressure wound therapy (postoperative day 29), granulation with wound contraction (postoperative day 69), and complete wound closure (postoperative day 90). The lower timeline summarizes key clinical events and stepwise, integrated wound and nutritional management.

### Nutritional management and outcomes

In the acute phase, total parenteral nutrition (TPN) was the mainstay. The nutritional support team was consulted early for multidisciplinary nutritional planning. Nutritional requirements were determined based on body weight and were adjusted according to stoma output, urine output, body weight, edema, blood glucose level, serum electrolyte levels, renal function, acid–base status, liver function, and C-reactive protein levels. Trace elements and vitamins (zinc; copper; selenium; folate; and vitamins D, B12, A, and E) were monitored weekly during periods of clinical instability or high stoma output. On POD 15, small amounts of enteral nutrition (EN) via a nasogastric tube were initiated and gradually increased. The stoma output initially exceeded 1800 mL/day but decreased to approximately 700–1000 mL/day with loperamide, codeine phosphate, tiropramide bromide, and proton pump inhibitors (PPIs). Oral intake was initiated on POD 42, targeting 25–35 kcal/kg/day. Serum albumin levels declined to <2.0 g/dL during the TPN period, then gradually increased after EN initiation to 2.2–3.0 g/dL, recovering to nearly 4.0 g/dL by the time of transfer.

The patient was transferred for rehabilitation on POD 120 and discharged with home PN. After discharge, she tolerated a regular oral diet and required PN (1000 kcal in 2000 mL) only on weekends, allowing her to leave home freely on weekdays. At the 1-year outpatient follow-up, her body weight was maintained at 54 kg (body mass index 22.8 kg/m^2^). Hemoglobin was 12.6 g/dL; serum iron, 101 µg/dL; vitamin B12, 301 pg/mL; zinc, 102 µg/dL; copper, 73 µg/dL; and magnesium, 1.7 mg/dL, indicating a stable nutritional status without anemia or evident deficiencies in the measured micronutrients. At 15 months postoperatively, she remained well with preserved activities of daily living, without central venous port infection, wound complications, or stoma-related problems.

## Discussion

The educational value of this case lies less in any single technique than in demonstrating how coordinated, phase-oriented wound and nutritional management achieved secondary-intention healing without reconstructive surgery despite the rare sequential convergence of perforated diverticulitis, septic shock, delayed NOMI, SBS, multidrug-resistant wound contamination, and severe wound dehiscence.

### NOMI diagnosis

Because NOMI may present with nonspecific symptoms and subtle imaging findings, it should remain in the differential diagnosis after septic shock requiring vasopressor support, even after transient hemodynamic stabilization^[^7^]^. In the present case, the initial event was perforated sigmoid diverticulitis with generalized fecal peritonitis (Hinchey grade IV) in an older patient receiving chronic corticosteroid and immunomodulatory therapy. This immunocompromised state may have contributed to the development of complicated diverticulitis. The subsequent NOMI was considered pathophysiologically interconnected with the initial perforation rather than an independent process because fecal peritonitis led to septic shock, vasopressor exposure, and a prolonged low-flow state predisposing to mesenteric hypoperfusion. Alternative etiologies were considered, including mesenteric vasculitis, autoimmune-mediated vascular injury, occlusive ischemia, progressive ischemic disease already present at the initial operation, and iatrogenic vascular injury. However, the absence of main-trunk occlusion on CT, preserved superior mesenteric artery pulsation, multifocal, segmental, and discontinuous involvement of the small intestine and colon, and predominantly peripheral vessel involvement on histopathology favored NOMI as the most likely diagnosis.

### Intensive wound management for SSI

The patient in the present case had multiple overlapping risk factors for SSI and wound failure, including advanced age, steroid or immunosuppressant use, heavy contamination during surgery, septic shock with vasopressor use, repeated laparotomies, and SBS-related malnutrition^[^8–11^]^. NPWT reduces local edema and promotes granulation through continuous wound exudate aspiration^[^12^]^ and avoids flap reconstruction in approximately 30% of complex wounds^[^13^]^. Povidone–iodine sugar promotes keratinocyte and fibroblast proliferation to support wound bed preparation^[^14^]^. Beyond its antiseptic effect, povidone–iodine sugar paste has been reported to activate keratinocyte and fibroblast functions, thereby promoting re-epithelialization and granulation-tissue formation^[^14^]^. In the present case, integrated wound management was implemented in phases. After repeated debridement and wound bed cleansing, NPWT was used primarily for exudate control, followed by intermittent biofilm removal. Following granulation and contraction, additional trafermin was administered to promote epithelialization. This integrated approach may enable secondary intention healing without skin grafting or re-suturing, despite extensive dehiscence exceeding 15 cm. Notably, NPWT was continued for a relatively prolonged period (4 weeks), longer than that typically reported for open-incision NPWT (median 4–7 days), owing to copious exudate and a large wound burden^[^15^]^.

### Nutritional management strategy for SBS

Nutritional management of SBS involves a staged transition from TPN-dominant in the acute phase to gradual escalation of EN during intestinal adaptation^[^16,17^]^. For high-output stoma (≥2 L/day), loperamide and opioid antidiarrheals can prolong intestinal transit time, and PPIs reduce small-intestinal fluid volume and electrolyte loss^[^18^]^.

In the present case, TPN was the mainstay in the acute phase, while EN was gradually increased with concomitant control of stoma output (700–1000 mL/day), stabilizing fluid and electrolyte balance and improving nutritional status, as reflected by rising serum albumin levels. For wound healing, 25–35 kcal/kg/day and 1.25–1.5 g/kg/day protein intake are recommended, as protein deficiency is associated with a 2- to 5-fold increase in SSI risk^[^3^]^. Accordingly, we pursued aggressive nutritional support with frequent caloric and protein adjustments, supplementation with trace elements (e.g., zinc and selenium) and vitamins A, C, and D, and amino acid administration.

### Limitations

First, this report describes a single retrospective case, and the approach may not be generalizable to other patients with NOMI, SBS, or severe SSI with wound dehiscence. Second, patient-specific risk factors, including long-term steroid and immunomodulator use, multiple laparotomies, and an extremely shortened residual bowel length, may have influenced the patient's management and prognosis. Third, the NPWT settings, local agents, and nutritional targets were based on prior reports and institutional experience rather than controlled trials. Finally, the follow-up period was relatively short, and formal functional and patient-reported outcomes, including quality of life, were not systematically evaluated. Further accumulation of cases and prospective studies are required.

## Conclusions

Delayed NOMI can progress after septic shock despite transient hemodynamic stability. Even when extensive bowel resection results in SBS, high-output stoma, and severe wound dehiscence, sustained recovery is possible through coordinated wound-bed preparation integrated with staged nutritional escalation and stoma-output control.

## Data Availability

Not applicable.
